# Measurable Residual Disease (MRD) by Flow Cytometry in Adult B-Acute Lymphoblastic Leukaemia (B-ALL) and Acute Myeloid Leukaemia (AML): Correlation with Molecular MRD Testing and Clinical Outcome at One Year

**DOI:** 10.3390/cancers15205064

**Published:** 2023-10-19

**Authors:** Riana van der Linde, Prudence N. Gatt, Sandy Smith, Marian A. Fernandez, Lachlin Vaughan, Emily Blyth, Jennifer Curnow, David A. Brown, Elizabeth Tegg, Sarah C. Sasson

**Affiliations:** 1 Department of Laboratory Haematology, Institute of Clinical Pathology and Medical Research, NSW Health Pathology, Westmead Hospital, Westmead, NSW 2145, Australia; lachlin.vaughan@health.nsw.gov.au (L.V.); elizabeth.tegg@health.nsw.gov.au (E.T.); 2 Faculty of Medicine and Health, Sydney Medical School, University of Sydney, Camperdown, NSW 2050, Australia; prudence.gatt@sydney.edu.au (P.N.G.); emily.blyth@health.nsw.gov.au (E.B.); jennifer.curnow@health.nsw.gov.au (J.C.); david.brown1@health.nsw.gov.au (D.A.B.); sarah.sasson@health.nsw.gov.au (S.C.S.); 3 Westmead Institute for Medical Research, University of Sydney, Sydney, NSW 2145, Australia; 4 Flow Cytometry Unit, Institute of Clinical Pathology and Medical Research, NSW Health Pathology, Westmead Hospital, Westmead, NSW 2145, Australia; sandy.smith@health.nsw.gov.au (S.S.); marian.fernandez@health.nsw.gov.au (M.A.F.); 5 Department of Haematology, Western Sydney Local Health District, Westmead Hospital, Westmead, NSW 2145, Australia; 6 Department of Clinical Immunology and Immunopathology, Institute of Clinical Pathology and Medical Research, NSW Health Pathology, Westmead Hospital, Westmead, NSW 2145, Australia

**Keywords:** measurable residual disease, minimal residual disease, MRD, AML, B-ALL, molecular MRD

## Abstract

**Simple Summary:**

Measurable residual disease monitoring is an important prognostic tool in haematological malignancies commonly performed by two modalities: flow cytometry and molecular methods. In this paper, we studied consecutive adult participants requiring flow cytometric measurable residual disease monitoring. This is one of the largest prospective Australian studies to date, providing a unique insight into the Australian context. We described five distinctive patterns associated with disease relapse and survival and also investigated correlation with molecular methods. Our results provide additional evidence that the correlation between molecular and flow cytometric methods is moderate in B-lymphoblastic leukaemia and poor in acute myeloid leukaemia. There was a strong association between flow cytometry results and relapse in acute myeloid leukaemia but less so for B-lymphoblastic leukaemia. Our novel data indicate that the pattern of change in measurable residual disease over time was associated with the risk of relapse, particularly in acute myeloid leukaemia and highlight the divergent ways measurable residual disease testing can be employed across different leukaemias.

**Abstract:**

Measurable residual disease (MRD) detected by flow cytometry (FC) is well established in paediatric B- lymphoblastic leukaemia (B-ALL) and adult chronic lymphocytic leukaemia (CLL), but its utility in adult B-ALL and adult acute myeloid leukaemia (AML) is less clear. In this prospective MRD study, one of the largest in Australia to date, we examined consecutive bone marrow aspirates from adult participants with B-ALL (*n* = 47) and AML (*n* = 87) sent for FC-MRD testing at a quaternary referral hospital in Sydney. FC-MRD results were correlated to corresponding Mol-MRD testing where available and clinical outcomes at three-month intervals over 1 year. B-ALL showed a moderate positive correlation (r_s_ = 0.401, *p* < 0.001), while there was no correlation between FC-MRD and Mol-MRD for AML (r_s_ = 0.13, *p* = 0.237). Five FC-MRD patterns were identified which had significant associations with relapse (X^2^(4) = 31.17(4), *p* > 0.001) and survival (X^2^(4) = 13.67, *p* = 0.008) in AML, but not in B-ALL. The three-month MRD results were also strongly associated with survival in AML, while the association in B-ALL was less evident. There was a moderate correlation between FC-MRD and Mol-MRD in B-ALL but not AML. The association of FC-MRD with relapse and survival was stronger in AML than in B-ALL. Overall, these findings suggest divergent utilities of FC-MRD in AML and B-ALL.

## 1. Introduction

Measurable residual disease (MRD) is defined as the presence of malignant cells below the morphological detection threshold of 5% [1]. An ideal MRD test should detect and quantify residual leukaemic cells with high sensitivity and specificity while also being reproducible between laboratories [2]. The ability of a test to identify leukaemic cells is reliant on the stage of treatment at collection, sample type, and quality [3,4]. Molecular testing and immunophenotyping using multiparameter flow cytometry (FC-MRD) are the two most common methods used to monitor MRD. Molecular MRD (Mol-MRD) involves identifying a specific molecular abnormality associated with the leukaemic clone, commonly performed by real-time quantitative polymerase chain reactions (RT-qPCR). The use of next-generation sequencing (NGS) for tracking MRD is becoming increasingly popular both in B lymphoblastic leukaemia (B-ALL) and acute myeloid leukaemia (AML) [5,6]. Mol-MRD has several advantages, including reproducibility and high sensitivity. The European Leukaemia Network (ELN) AML guidelines recommend that Mol-MRD should be reported to at least 10^−3^%, and in practice, sensitivity usually ranges between 10^−3^ to 10^−6^% [5,7]. A caveat of Mol-MRD testing is that only ~40–50% of AML cases and ~90% of B-ALL cases have an appropriate molecular marker. Additionally, Mol-MRD markers present at diagnosis may be lost over time because of disease heterogeneity and clonal evolution [8,9,10]. Additionally, in AML, most Mol-MRD markers are associated with favourable-risk disease, so intermediate- and adverse-risk patients are often monitored using flow cytometry [7]. When using NGS, 2021 European Leukaemia Net (ELN) AML guidelines recommend that all mutations should be included, except for known germline mutations, mutations associated with signalling pathways, and age-related clonal haematopoiesis [5]. It is still unclear how NGS may contribute to MRD monitoring of B-ALL [6].

MRD monitoring by flow cytometry is currently a cornerstone in the management of AML and is commonly used in B-ALL [10,11]. Healthy bone marrow cells show reproducible patterns of maturation, and an expert understanding of normal bone marrow maturation and the impact of treatment on normal maturation patterns are critical to the interpretation of MRD measurement by flow cytometry [12,13]. The principle of FC-MRD monitoring is based on the evaluation of the leukaemia-associated immunophenotype (LAIP) and determining the “different from normal” (DfN) maturation patterns [7,13,14]. A combination of these two methods is often utilised in clinical practice and is recommended by the ELN [1,5,15,16].

Flow cytometry offers several advantages over Mol-MRD, such as its applicability to the majority of AML and B-ALL cases, a shorter turnaround time, lower cost, and the ability to provide information on both normal and leukaemic populations [8]. Flow cytometry is also well positioned for the identification of novel therapies, both those currently under investigation and potential future targets. Furthermore, FC-MRD can facilitate disease monitoring in a large group of intermediate- and adverse-risk AML patients that do not have molecular MRD targets. FC-MRD usually detects MRD down to a level of 0.01% to 0.1% of CD45^+^ cells but can be as sensitive as RT-qPCR [4,7]. Limitations of flow cytometry can be categorised into technical and analytical aspects. Technical aspects encompass aspects of sample quality like cell viability, treatment stage, hemodilution, and hypoplasia [14,17].

Analytical factors, including the wide variety of antibody clones and reagents, lead to interlaboratory method heterogeneity and lack of standardisation. Recent guidelines are aiming to address this issue [12]. Treatment-related bone marrow regeneration aberrancies also contribute to difficulties in FC-MRD analysis [8,18]. 

Monitoring of MRD in B-ALL, by either flow cytometry or molecular methods, is well established as a powerful prognostic tool to guide treatment decisions. It was initially utilised in paediatric B-ALL but has also proven effective in adult B-ALL. Achieving MRD-negative status earlier in the treatment course is associated with superior outcomes [11,13,19]. This is true for standard and high-risk B-ALL, including both Philadelphia chromosome-positive and negative B-ALL [8,20,21]. The National Comprehensive Cancer Network and the European Society for Medical Oncology clinical practice guidelines both advocate for MRD quantification as the standard of care [19].

The utility of MRD monitoring in AML is gaining recognition, particularly for favourable and intermediate-risk groups. There may also be benefits for adverse-risk patients, although information in this context is limited [14,22]. MRD monitoring following AML induction therapy is being used in clinical trials to guide treatment [5,9,17,23]. Recently, both the Food and Drug Administration (FDA) and the ELN have developed guidelines for reporting MRD, emphasising the need to report the limit of detection (LOD) and avoiding the selection of markers that may detect clonal preleukaemic populations [9,17]. These guidelines also underscore the importance of clinically validating MRD results. The current ELN guidelines propose a cutoff of 0.1% of CD45^+^ cells for AML MRD, supported by published data while acknowledging that lower levels of MRD, ranging from 0.01% to 0.1% CD45+ cells, may still hold clinical significance [5,14,24]. 

Very few studies have assessed the agreement between MRD monitoring by flow cytometry and molecular methods in AML, particularly in a prospective manner. Ouyang et al. [25] compared FC-MRD with RT-qPCR for core-binding factor (CBF) AML and found weak agreement between the two methods (k = 0.151). Another study comparing CBF AML with FC-MRD found a concordance of 67%, with 24/74 samples being discordant [26]. A study of *NPM1* in AML compared FC-MRD to RT-qPCR and found 57% concordance [27]. Conversely, multiple studies comparing molecular and FC-MRD methods in B-ALL have shown a good correlation [28,29,30]. 

FC-MRD is correlated with patient outcomes in B-ALL at different treatment timepoints and for different disease subtypes [11]. In AML, the association between FC-MRD positivity levels and variability of expression over time and clinical outcome is not as well established. Here, we analysed consecutive adult cases of AML and B-ALL undergoing FC-MRD testing and related these findings to Mol-MRD testing, disease relapse, and one-year survival in a single-centre prospective study. To our knowledge, few studies have directly compared the performance of FC-MRD to Mol-MRD across both adult B-ALL and AML. Furthermore, we investigated whether FC-MRD at a single timepoint is related to survival and whether the results over time are related to this clinical outcome. 

## 2. Materials and Methods

### 2.1. Study Design 

We studied 134 consecutive bone marrow aspirates from adult participants with B-ALL (*n* = 47) and AML (*n* = 87) sent for FC-MRD testing at NSW Health Pathology (NSWHP), Institute of Clinical Pathology and Medical Research (ICPMR), Westmead Hospital. Our centre is a major quaternary referral and bone marrow transplant centre that serves Sydney, regional New South Wales (NSW), and the Australian Capital Territory (ACT). Data were collected over a two-year period between January 2021 and December 2022. Participants were enrolled in the study if they had an FC-MRD test during the first year (2021). Longitudinal follow-up data on this group were then collected at three-month intervals during 2021 and 2022 so that all participants were followed for a maximum of one year. Demographic and clinical information was collected, including age, sex, underlying diagnosis, treatment, flow cytometry, cytogenetic, and molecular results. The data were stored in a soft-copy format on a secure password-protected computer server with limited access according to NSW Health policies. This study was approved by the Western Sydney Local Health District Human Research Ethics Committee (2020/ETH01526).

### 2.2. Sample Preparation, Instrument Setup, and Cell Acquisition

Samples were collected in Roswell Park Memorial Institute media (RPMI), and 90% of samples were processed within 12–24 h, 98% within 48 h, and the remaining samples within 72 h. Samples were prepared using commercial ammonium chloride 9% lysing solution (Kinetik Pty Ltd., Sydney, NSW, Australia). Events were acquired using the Beckman Coulter Gallios instrument (Beckman Coulter Life Sciences, Brea, CA, USA). A target value of 500,000 CD45^+^ cells was acquired for each MRD assessment, with a minimum of 100,000 CD45^+^ cells required for FC-MRD reporting. FC-MRD was expressed as a percentage of CD45^+^ cells.

### 2.3. Immunophenotypic MRD Analysis 

AML FC-MRD analysis was performed using two panels: (i) a general myeloid panel and (ii) a monocytic panel. The B-ALL MRD was performed using one panel. The monoclonal antibodies used in each tube are described in Supplementary Table S1.

Flow cytometric data were analysed using Kaluza software version 2.2.1 (Beckman Coulter Life Sciences). The leukaemia-associated immunophenotype (LAIP) was established at diagnosis using sequential Boolean gating and bivariate plots. Subsequent bone marrow samples were assessed for the presence of MRD by flow cytometry on the basis of the presence of the LAIP and also reviewed for any abnormal populations that were different from normal because of the expression intensity of normal markers and/or expression of aberrant markers. MRD analysis was performed using both the reference to the LAIP and the difference from normal and in line with European Leukaemia Network guidelines [5,9]. Briefly, the blast region was identified (CD45 dim/low side-scatter) and underwent sequential Boolean gating. A population was identified as MRD if the expression profile was similar to the LAIP or showed aberrant features when compared with normal maturation or regenerating bone marrow. A minimum of 500,000 CD45^+^ events was acquired, and the MRD percentages were calculated using CD45^+^ events as the denominator. The limit of detection was determined to be 20 clustered within the MRD gate. MRD was deemed positive when it was >0.01% of CD45^+^ events. Indeterminate results were reported when MRD was detected below 0.01% of CD45^+^ cells or a population was detected that had a similar expression profile to the LAIP but also significantly overlapped with normal bone marrow cells. A patient was deemed to have relapsed if the residual disease was >5% of CD45^+^ cells. An example of an MRD positive and MRD negative case can be seen in Supplementary Figures S1 and S2.

### 2.4. Molecular MRD Analysis

The quantitative *RUNX1-RUNX1T1* [t(8;21)] and *BCR-ABL1* [t(9;22)] analyses were performed in the NSW Health ICPMR Westmead Hospital Diagnostic Molecular Laboratory using an intercalating dye, Syto9, to detect and quantitate the amount of transcript during RT-qPCR. The result was presented as a ratio of *RUNX1-RUNX1T1* or *BCR/ABL1* transcript divided by *ABL1* transcript. The quantitative *NPM1* mutational analysis was performed at Royal Prince Alfred Hospital, NSWHP, Sydney, using the Ipsogen *NPM1* Mut A MutaQuant Kit (Qiagen, Venlo, The Netherland). The *CBFB-MYH11 A* [inv(16/t(16;16)] mutational analysis was performed at Peter MacCallum Cancer Centre, Melbourne, using the Ipsogen *CBFB-MYH11* A kit (Qiagen). RT-qPCR for immunoglobulin gene rearrangements was referred to the Children’s Cancer Institute Australia, UNSW, Sydney. MRD markers were developed from diagnostic or relapsed patient samples using conventional PCR amplification and Sanger sequencing, identifying variable gene rearrangements and aberrations with a sensitivity of approximately 10%. Mol-MRD testing was performed by RT-qPCR on DNA isolated from remission sample(s) to measure the prevalence of the leukaemic clone previously detected in the reference sample obtained at leukaemic diagnosis or relapse. Assay results were interpreted according to the guidelines established by the EuroMRD group [31].

### 2.5. Data Analysis

The results from FC-MRD and Mol-MRD were correlated using Spearman’s rank correlation, measuring the direction and strength of association between ranked variables. Spearman correlation compared the ranks of values of two variables instead of the values themselves. The ranked correlation was selected because our MRD data were nonparametric, being skewed to the right and containing numerous zeros. The ranked data were represented in a scatterplot, with the negative values having the highest ranking. The scatterplots can be divided into four quadrants, with the concordant negative results in the top right corner, the concordant positive in the bottom left, and the discordant results in the top left and bottom right corners. 

FC-MRD analysis for each three-month timepoint was analysed using descriptive statistics, and the association with survival was determined by the Mann–Whitney U test. The association between FC-MRD and Mol-MRD, ELN risk, treatment modalities, MRD patterns, and patient outcome was evaluated using an independent X^2^ (Chi-squared) test of association. All statistical analyses and correlation graphs were performed using Jamovi software (Version 2.6) [32,33]. A *p*-value of <0.05 was considered statistically significant. Sankey plots were used to illustrate the relationship between FC-MRD patterns, ELN risk, relapse, and survival [34].

## 3. Results

### 3.1. Patient Demographics and Treatment

#### 3.1.1. B Lymphoblastic Leukaemia (B-ALL)

B-ALL group comprised 47 participants (35% of the cohort), with 223 discreet FC-MRD results. The median patient age was 51 years, with 62% being male (*n* = 29). At study enrolment, 68% had received chemotherapy (*n* = 32), 19% had an allogeneic stem cell transplant (alloSCT) (*n* = 9), and 4% were under surveillance after completing treatment (*n* = 2). Chemotherapy consisted of multimodal regimens (*n* = 19, 59%), blinatumomab (*n* = 3, 9%), and maintenance (*n* = 10, 32%). A total of 44% of alloSCT participants received reduced intensity conditioning (*n* = 4) and 56% myeloablative conditioning (*n* = 5). Donor types were divided into matched unrelated (*n* = 3, 33%), haploidentical (*n* = 2, 22%), and sibling (*n* = 4, 45%). In addition, 13% more participants had an alloSCT during the study period (*n* = 6), 11% (*n* = 5) of participants had clinical relapse during the study period, and 9% (*n* = 4) died.

At baseline, 26% of B-ALL participants had a positive FC-MRD (*n* = 12), 70% negative (*n* = 33), and 4% indeterminate (*n* = 2). A total of 76% had a measurable Mol-MRD marker, 53% BCR-ABL1, [t(9;22)] (*n* = 19), and 47% had the *IGH* gene rearrangement (*n* = 17). There were 120 discreet Mol-MRD results (Table 1).

#### 3.1.2. Acute Myeloid Leukaemia (AML)

AML comprised 87 participants (65% of the cohort), with 259 discreet FC-MRD results. Their median age was 60, with a slight male predominance (52%) in keeping with the literature [35]. Distribution according to ELN risk stratification was as follows: favourable risk 29% (*n* = 25), intermediate risk 26% (*n* = 23), and adverse risk 33% (*n* = 29). A total of 7% (*n* = 6) could not be classified because of the lack of information or because they had secondary AML transformed from a myeloproliferative neoplasm. At the time of study enrolment, the participants had received the following treatment modalities: Chemotherapy 56% (*n* = 49), alloSCT 33% (*n* = 29), or surveillance 8% (*n* = 8). Of those receiving chemotherapy, 65% received intensive regimens (*n* = 32), 10% received hypomethylating-based regimens (*n* = 5), and 25% low-dose cytarabine-based regimens (*n* = 12). Furthermore, 66% of those receiving alloSCT had reduced-intensity conditioning (*n* = 19) and 34% myeloablative conditioning (*n* = 10), while 48% had a matched unrelated donor (*n* = 14), 38% a haploidentical donor (*n* = 11), and 14% a sibling donor (*n* = 4). Eleven participants (23%) received an alloSCT during the one-year follow-up period, while 10% (*n* = 9) of participants relapsed during the study period and 16% (*n* = 14) died.

Baseline FC-MRD results were positive in 51% (*n* = 44), negative in 26% (*n* = 23), and indeterminate in 23% (*n* = 20). A total of 33% of cases had a measurable Mol-MRD marker, with 100 discreet molecular results. The molecular subtypes were as follows: *RUNX1-RUNX1T1*, [t(8;21)] in 26% (*n* = 8) *CBFB-MYH11,* [inv(16)] in 8% (*n* = 2), *NPM1* mutations in 62% (*n* = 18), and *BCR-ABL1*, [t(9;22)] in 4% (*n* = 1) (Table 2).

**Figure 1:**
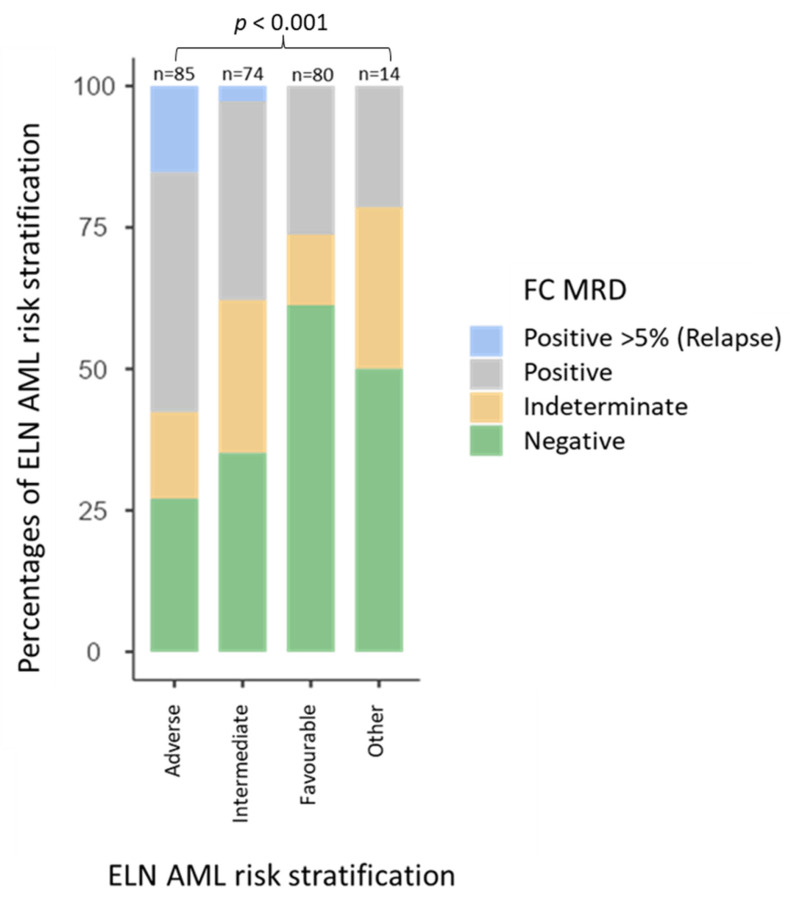
Flow cytometric measurable residual disease (FC-MRD) results associated with European Leukaemia Network (ELN) acute myeloid leukaemia (AML) risk stratification groups. A total of 59% of adverse-risk AML MRD results were positive, including relapsed patients, 27% of MRD results in intermediate-risk AML were indeterminate, and 60% of MRD results in favourable-risk AML were negative.

There was a strong association between the FC-MRD results and ELN risk (X^2^(9) = 41.72, *p* < 0.001). At any timepoint, a positive FC-MRD result (positive or relapse) was predominantly associated with the adverse-risk group, 53% (*n* = 34). Indeterminate FC-MRD results were more commonly associated with intermediate-risk AML (46%, *n* = 6). FC-MRD was negative in 39% of the favourable-risk AML (Figure 1).

### 3.2. Comparison of FC-MRD and Mol-MRD Results

#### 3.2.1. B-ALL

**Figure 2:**
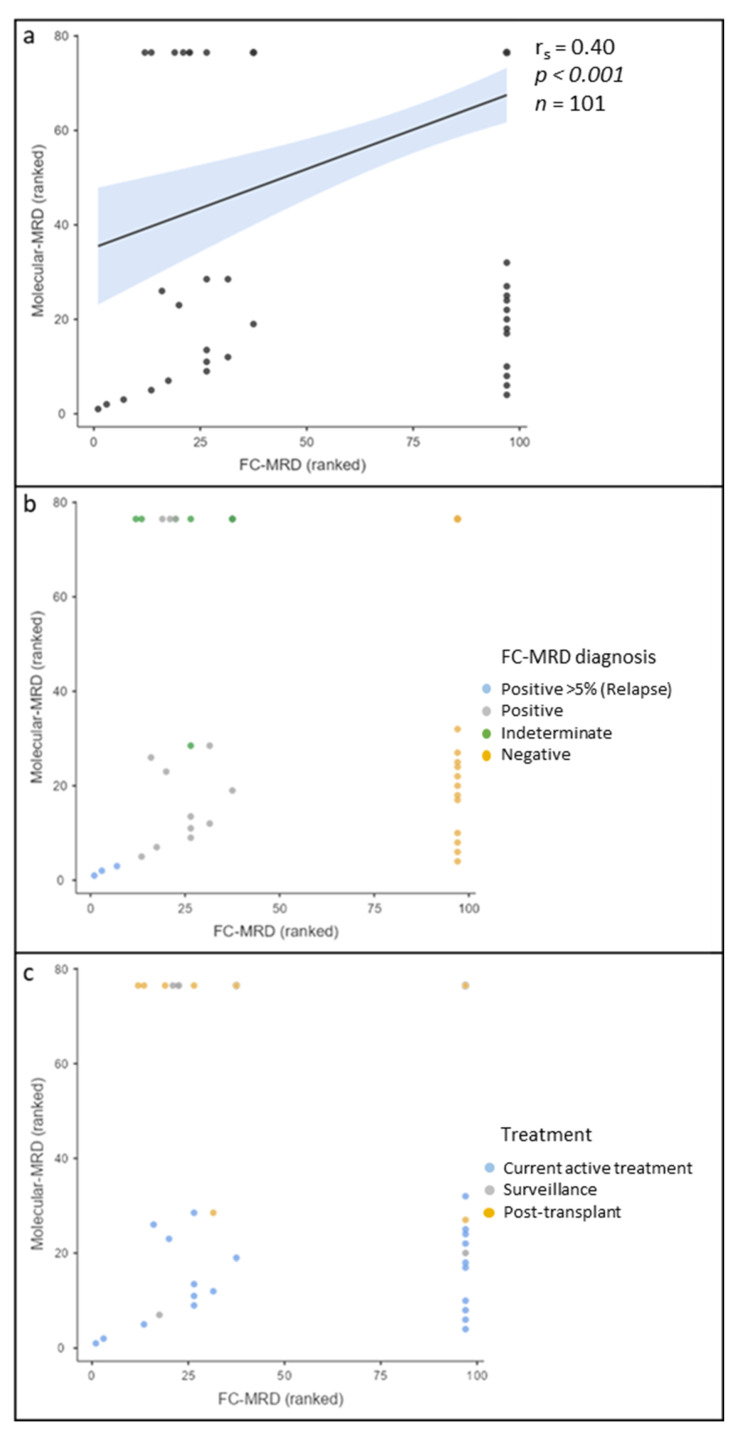
Scatterplot of ranked B-lymphoblastic leukaemia (B-ALL) measurable residual disease (MRD) results showing correlation of flow cytometric (FC) MRD and molecular MRD (Mol-MRD). Top right: Concordant negative results. Bottom left: Concordant positive results. Bottom right: Negative FC MRD, but positive molecular MRD. Top left: Molecular MRD negative, but positive FC MRD. (**a**) Moderate positive correlation between FC-MRD and Mol-MRD. (**b**) Grouped according to FC-MRD results, showing FC-MRD > 5% is consistently concordant with Mol-MRD. Indeterminate results were predominantly discordant. MRD < 5% were variably concordant and discordant. (**c**) Grouped according to treatment modality. The post-transplant and surveillance cohorts were consistently discrepant, predominantly FC-MRD positive and Mol-MRD negative. Active treatment varied between concordant results and discordant, with discordant results predominantly Mol-MRD positive and FC-MRD negative.

There were 101 discreet FC-MRD results with a concurrent Mol-MRD result. There was a moderate positive correlation between FC-MRD and Mol-MRD (r_s_ = 0.401, *p* < 0.001). Figure 2 shows the correlation of the ranked data. When grouping the data into positive, negative, or indeterminate FC-MRD, it was observed that the discrepant data with negative Mol-MRD and positive FC-MRD were more frequently classified as indeterminate. (Figure 2b). And when grouped according to treatment modalities, the discrepancies were predominantly associated with the post-transplant participants (Figure 2c).

We further investigated the impact of a 0.01% versus a 0.1% CD45^+^ cell threshold for FC-MRD. There was a strong association between FC-MRD and Mol-MRD results (X^2^(3) = 24.28, *p* < 0.001) when using a 0.01% cutoff. When Mol-MRD was positive, 40% of tests were also positive for FC-MRD (Table 3). The association between the two treatment modalities was not improved by increasing the FC-MRD cutoff to 0.1% of CD45^+^ cells (X^2^ = 14.6, *p* < 0.001). A large proportion of indeterminate results (*n* = 8/9) were below 0.1% (range = 0.01–0.4, median = 0.02), and most of these were negative by Mol-MRD.

#### 3.2.2. AML

**Figure 3:**
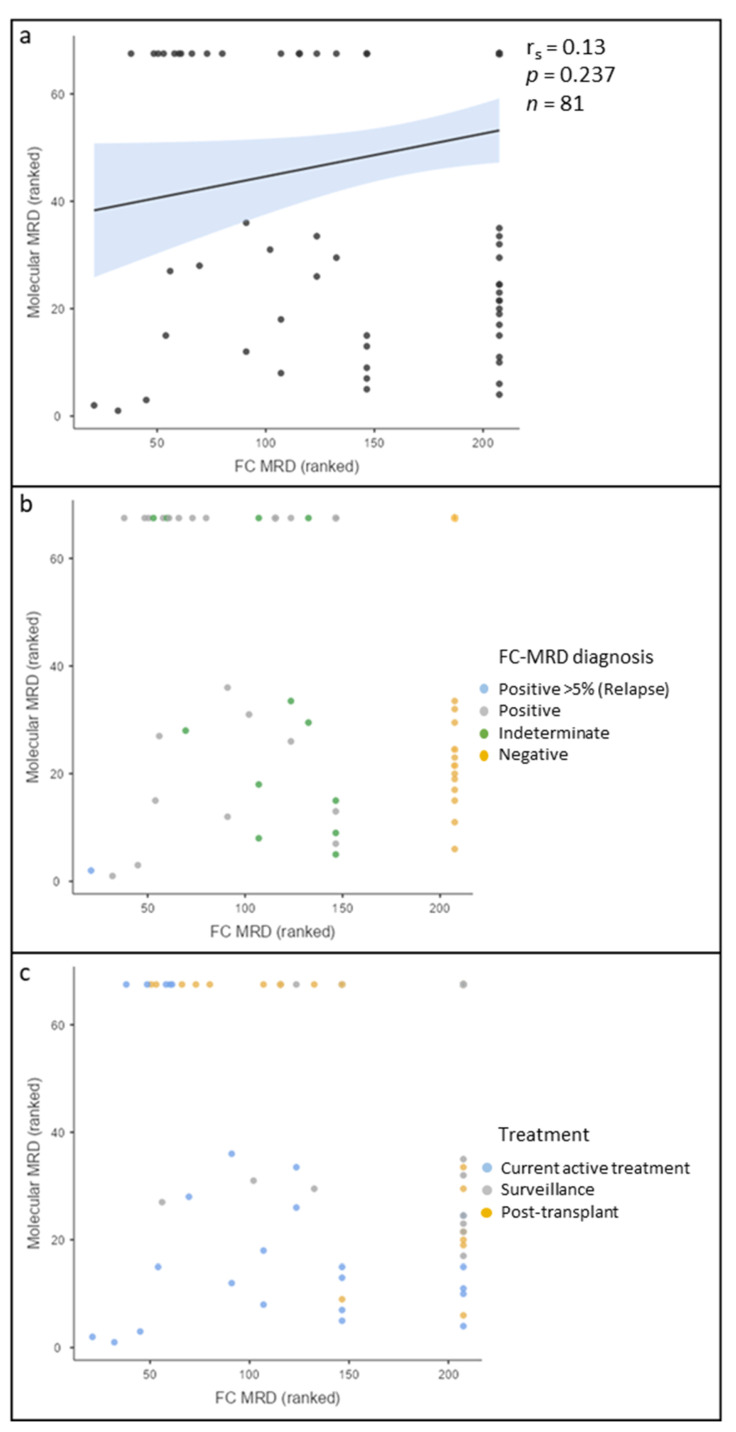
Scatterplot of ranked acute myeloid leukaemia (AML) measurable residual disease (MRD) results showing correlation of flow cytometric (FC) MRD and molecular (Mol) MRD. Top right: Concordant negative results. Bottom left: Concordant positive results. Bottom right: Negative FC-MRD, but positive Mol-MRD. Top left: Mol-MRD negative, but positive FC-MRD. (**a**) No correlation between FC-MRD and Mol-MRD. (**b**) Grouped according to FC-MRD results, showing FC-MRD > 5% is consistently concordant with Mol-MRD. Discordant results were associated with MRD < 5% and indeterminate results. (**c**) Correlation graph grouped according to treatment modality. The post-transplant cohort was consistently discrepant, either FC-MRD positive and Mol-MRD negative or FC-MRD negative and Mol-MRD positive. Active treatment generally had a good correlation, while surveillance marrows showed some discordance, predominantly Mol-MRD positive and FC-MRD negative.

There were 82 discreet FC-MRD results that had a concurrent Mol-MRD result. Figure 3 shows the correlation of the ranked data for FC-MRD and Mol-MRD. There was no evidence of a correlation between FC-MRD and Mol-MRD (r_s_ = 0.13, *p* = 0.237, *n* = 81). No clear relationship was observed when grouping the data into positive, indeterminate, and negative (Figure 3b). However, when separating the data into treatment groups, it was observed that, similarly to B-ALL, the post-transplant cohort results were consistently discrepant (Figure 3c).

There was a trend towards an association between FC-MRD and Mol-MRD results (X^2^(3) = 6.32, *p* = 0.097) when using a 0.01% cutoff for FC-MRD, but this did not reach statistical significance. (Table 4). The association between the two treatment modalities was not improved by increasing the FC-MRD cutoff to 0.1% (X^2^ = 0.25, *p* = 0.883). Further interrogation of the treatment categories demonstrated an association between FC-MRD and Mol-MRD within the active treatment group (X^2^(2) = 8.01, *p* = 0.018). There was no evidence of an association between flow cytometry and molecular results for the post-transplant group (X^2^(2) = 4.10, *p* = 0.128) or the surveillance groups (X^2^(2) = 1.27, *p* = 0.528) (Supplementary Table S2).

### 3.3. Longitudinal FC-MRD Patterns 

#### 3.3.1. B-ALL

**Figure 4:**
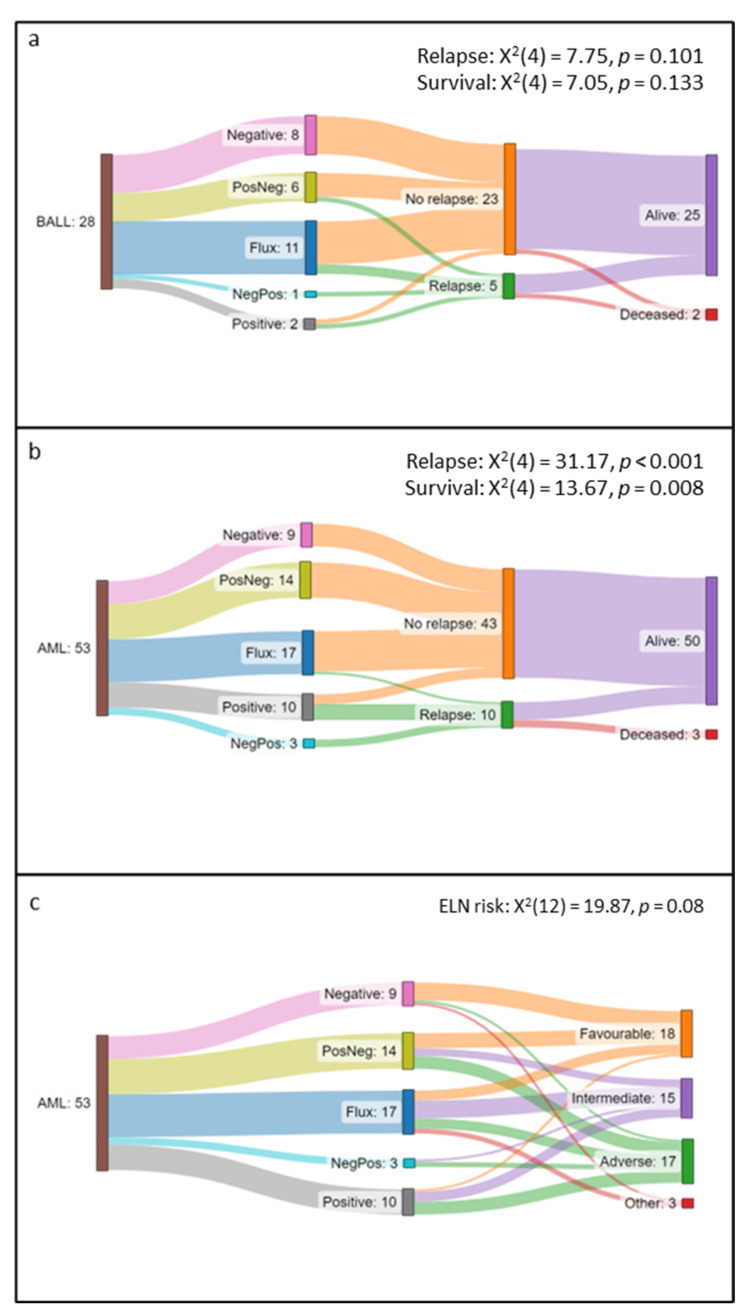
Sankey graphs showing association between measurable residual disease (MRD) patterns, European Leukaemia Network (ELN) risk stratification, relapse, and survival data for 53 acute myeloid leukaemia (AML) participants and 28 B-lymphoblastic leukaemia (B-ALL) participants with ≥3 MRD results over one-year follow-up. Pathways: *Negative:* all MRD results negative; *PosNeg:* results converting from positive at baseline to become negative; *Flux:* variation of results between positive, negative, and indeterminate; *Positive:* all MRD results positive; *NegPos*: the negative result at baseline, with a later positive result. (**a**) B-ALL: No participants in the Negative pathway relapsed or died. One patient from the PosNeg, two from the Flux, one from NegPos, and one from the Positive pathways relapsed. Two participants died, one from the Positive pathway and the second from the Flux pathway, due to transplant complications. (**b**) AML: No participants in the Negative and PosNeg pathways relapsed or died. All patients in the NegPos and seven participants in the Positive pathways relapsed. One patient from the Flux pathway relapsed and subsequently received a transplant. Three participants died, all originating from the Positive group. (**c**) The Negative group were predominantly associated with favourable-risk AML. The PosNeg and Flux groups were evenly divided between AML risk groups. NegPos and Positive patterns were mostly associated with intermediate or adverse risk AML.

We aimed to describe the longitudinal patterns of FC-MRD over the one-year follow-up period. In B-ALL, 28 participants had three or more FC-MRD results during the follow-up period and were included in this analysis. We divided these participants into five patterns: (1) Negative (*n* = 8) all FC-MRD results are negative, (2) Positive to Negative (PosNeg; *n* = 6) FC-MRD converted from positive to negative over the study period, (3) Flux (*n* = 11) FC-MRD fluctuated between positive and negative over the study period, (4) Negative to Positive (NegPos; *n* = 1) FC-MRD converted from negative to positive over the study period, and (5) Positive (*n* = 2) all FC-MRD results were positive during the study period. Figure 4a shows the association between the patterns, relapse, and survival. Five cases demonstrated overt clinical relapse during the one-year follow-up period from four different groups: PosNeg (*n* = 1), Flux (*n* = 2), NegPos (*n* = 1), and Positive (*n* = 1). Two participants died: one from the Positive group, who also relapsed, and a second from the Flux group, who died from post-alloSCT complications. In general, the Flux group showed very low FC-MRD, mostly <0.1%, with all participants being alive after twelve months and only two having relapsed. There was no evidence of an association between B-ALL patterns and relapse (X^2^(4) = 7.75, *p* = 0.101) or survival (X^2^(4) = 7.05, *p* = 0.133).

#### 3.3.2. AML

Fifty-three participants had three or more FC-MRD results during the one-year follow-up period and were included in the longitudinal pattern analysis. AML participants’ results were grouped as previously described for B-ALL. The distribution of participants was: Negative *n* = 9, PosNeg *n* = 14, Flux *n* = 17, NegPos *n* = 3, and Positive *n* = 10. Figure 4b,c shows the relationship between the patterns, ELN risk stratification, relapse, and survival. The majority of relapsed participants (*n* = 10) derived from the NegPos and Positive groups, with the notable exception being one patient from the Flux group who subsequently underwent a transplant. There were three deaths, all from the Positive group. 

There was strong evidence of an association between FC-MRD patterns and clinical relapse (X^2^(4) = 31.17(4), *p* > 0.001) and survival (X^2^(4) = 13.67, *p* = 0.008). There was a trend towards an association between FC-MRD patterns and ELN risk stratification (X^2^(12) = 19.87, *p* = 0.08).

### 3.4. FC-MRD Association with Clinical Relapse and Death 

#### 3.4.1. B-ALL

Six B-ALL participants relapsed during the study; one had a positive FC-MRD in the three months preceding the relapse, and two had a negative FC-MRD in the preceding three months, with one also having a negative Mol-MRD test. Three participants had no FC-MRD conducted in the preceding three months, and one of them had a positive Mol-MRD during the same period. 

We examined whether the level of FC-MRD at any timepoint was associated with one-year mortality. At three months, the median FC-MRD level was greater in the participants who did not survive to one year compared with survivors, with the medians being 0.17% and 0%, respectively (U = 4, *p* = 0.014). However, this association was not significant at baseline, six months, or one year (Table 5).

#### 3.4.2. AML

Nine AML participants relapsed during the study; five had a positive FC-MRD in the preceding three months, two had an indeterminate FC-MRD result, one had a negative FC-MRD result, and one had no FC-MRD result.

There was evidence of an association between the level of FC-MRD present at each of the study timepoints and survival at one year, with non-survivors having higher levels of FC-MRD (Table 6). The strongest association was at study enrolment, where participants who survived to one year had a lower FC-MRD compared with non-survivors (0.03% compared to 1.39% of CD45^+^ cells, U = 118, *p* < 0.001).

## 4. Discussion

The direct comparison of MRD results deriving from flow cytometric and molecular methodologies has only been addressed in a limited number of published reports. Additionally, few of these studies investigated this in a prospective design and also interrogated the divergent roles that MRD monitoring may play in adult B-ALL and AML. 

Available reports comparing these two modalities in B-ALL show a good correlation between the two methods, ranging between 86 and 98% [28,29,30]. Our cohort only showed a moderate correlation between the two methods but a very strong association at a 0.01% cutoff. The reasons for the poorer correlation when compared with the literature are unclear but may be related to different treatment regimens, with our data showing greater discrepancies in the post-transplant cohort. An additional cause for divergent results between MRD assays may be due to differences in test targets (i.e., surface proteins vs. molecular targets) and the related specificity and sensitivity. Sample consistency may also have played a role, with the first bone marrow aspirate draw routinely being sent for mol-MRD and the subsequent draw sent for FC-MRD, leading to the potential for discrepancies to occur across FC-MRD and Mol-MRD samples. To assess this, estimates of haemodilution, such as neutrophil percentage, myeloid precursors, B-cell precursors, mast cells, and nucleated red blood cells, can be made sequentially in bone marrow aspirate draws [36]. However, as this cannot be addressed retrospectively, it will be an area of ongoing investigation. 

The literature comparing FC-MRD and Mol-MRD in AML reports a relatively poor correlation between the two methods and has largely been limited to a single molecular target Shang et al. Authors of [37] compared MFC-MRD to PCR for t(8;21), with the overall correlation being less than 50%. Other studies have observed similar results, with concordance ranging from 15 to 67%, and the postinduction timepoint typically exhibiting the weakest correlation [25,27,38,39]. Our study showed no correlation or association between FC-MRD and Mol-MRD. It should be noted that there is evidence of the prognostic value of discrepant results in AML, with outcomes being intermediate between those of patients with concordant MRD negative or MRD positive results [40,41,42,43]. 

The lack of correlation between methodologies in our AML cohort may be related to several aspects. Firstly, discrepancies with Mol-MRD being positive and FC-MRD negative are likely due to the increased sensitivity of molecular tests [2]. This is supported by our results, where very low-level Mol-MRD of <0.01% was detected when FC-MRD was negative. However, it is important to note that low-level Mol-MRD does not always correspond to an increased risk of clinical relapse. The ELN guidelines define Mol-MRD with low copy numbers (MRD-LCN) as a transcript level < 1–2% with a <1-log change between any two positive samples at the end of treatment [5]. Prognostic studies have demonstrated that the persistence of MRD-LCN in *NPM1* AML is associated with a very low risk of relapse [44]. Similar studies for other molecular markers are not yet available.

We propose that the group of patients with positive FC-MRD but negative Mol-MRD is likely to be more diverse. In the context of AML specifically, potential explanations include the tendency of AML to exhibit clonal evolution, bone marrow regeneration following treatment, and/or the presence of preleukaemic myeloid populations that complicate the interpretation of MRD results [1,27,37]. Interestingly, most of the discrepancies between methodologies occurred within the post-transplant cohort in both AML and B-ALL. The reason for this is uncertain but suggests a more significant treatment impact on the bone marrow milieu, including regenerating precursor populations that may overlap more significantly with the LAIP, complicating FC-MRD interpretation.

Our AML cohort showed a strong association between FC-MRD results and ELN risk, with adverse risk having a larger proportion of FC-MRD positive results, intermediate risk having more indeterminate results, and favourable-risk groups having the largest proportion of negative results. This is in keeping with the literature [45].

FC-MRD is known to have false-positive and false-negative results due to the limitations stated previously. One way of reducing the impact of this is by doing serial MRD testing [3]. Statistical metrics of MRD analysis suggest that serial monitoring of MRD would be more relevant to clinical outcomes in both FC-MRD and Mol-MRD analyses, but despite this, not many FC-MRD studies have addressed this. [3] A study by Liu et al. showed that sequential MRD monitoring had a greater prognostic impact compared with MRD values at specific timepoints [24]. A novel aspect of this study is that we describe five distinct FC-MRD patterns. The AML cohort showed a significant association between the pattern of MRD expression over time and outcomes with regard to relapse or survival. The association between B-ALL patterns, relapse, and survival did not reach statistical significance, possibly due to the low numbers of relapse and death in this group. Negative or decreasing FC-MRD over one year were associated with favourable outcomes. Persistently positive or increasing FC-MRD was linked to poor outcomes. While these results are somewhat expected, the group with fluctuating results were interesting. The MRD in the “Flux” group was in the low range, with the medians for AML and BALL being 0.02% and <0.01% of CD45^+^ cells, respectively. Only one patient in the AML “Flux” group relapsed at six months, and all were alive at one year. Two participants from the B-ALL “Flux” group relapsed, and all were alive at one year. These novel data suggest that consecutive FC-MRD results with increasing MRD are more predictive of clinical relapse than a single positive FC-MRD result, and this may be particularly relevant in AML. 

Previous work has demonstrated that MRD measurement is prognostic in favourable and intermediate-risk participants, while studies vary on the significance of adverse risk AML [22,24,45]. ELN stratification trended towards an association with FC-MRD patterns in AML. Favourable-risk AML were more prominent in the negative/reducing patterns. Intermediate-risk AML was predominantly associated with fluctuating patterns, while positive or increased FC-MRD patterns were more prominently associated with adverse-risk AML. The association between ELN risk stratification and FC-MRD patterns was less pronounced than with relapse and survival. Importantly, this implies that the longitudinal patterns of FC-MRD are significant and that relapse and survival were not determined only by baseline risk stratification. 

In B-ALL, FC-MRD has been shown to be clinically significant with regard to patient outcomes in adult participants for standard-risk, high-risk, Philadelphia chromosome-positive, and post-transplant participants [8,11,13,20,21]. Similarly, for AML, it has been reported that FC-MRD is associated with clinical outcomes with AML in several different situations, including after induction, early consolidation, and pretransplant [10,14,22,24,37].

Our data highlight the different utilities of FC-MRD in B-ALL and AML. In B-ALL, the association was significant only at the three-month timepoint. The lack of significance at other timepoints may be influenced by an overall low death rate. However, it is important to note that in B-ALL, changing treatment based on MRD results is currently considered best practice [8,46]. Therefore, intervention after a positive MRD result may also explain the lack of a clear association between the FC-MRD results at specific timepoints and disease relapse. However, this needs to be confirmed with larger cohorts and longer follow-ups. 

We report a strong association between FC-MRD levels and one-year survival in the AML cohort. The level of FC-MRD was consistently higher at every timepoint in the group of non-survivors. This is probably influenced by several factors, including risk stratification (almost all cases were either intermediate or adverse risk) and treatment, with >50% of AML participants not receiving curative therapy. What remains unclear is whether an intervention would change the outcome of patients and, if so, what the ideal time of intervention would be. We are currently involved in a larger multicentric study that addresses some of these issues (https://trials.cancervic.org.au/details.aspx?ID=vctl_actrnactrn12621000439842 (accessed on 3 August 2023)). 

The limitations of our study are that it was a single-centre study with a relatively short follow-up period. However, to date, our study is one of the biggest prospective studies of this kind, providing valuable data and additional insight in an Australian context. We have ethics approval to continue data collection for at least two years, with the potential to continue for longer if needed. We plan on providing an updated report at the two-year timepoint. Another shortcoming identified during our study is the lack of data on the haemodilution of samples, which may explain some of the discrepancies between FC-MRD and Mol-MRD results. This can be addressed in future work. In ongoing studies, it may be useful to focus on specific treatment regimens and treatment stages, such as post-transplant or postconsolidation. Studies that additionally incorporate NGS and an extended follow-up period will also be of great interest. 

## 5. Conclusions

In adults, the correlation between FC-MRD and Mol-MRD is fair for B-ALL but absent for AML, likely reflecting a greater disease complexity, including heterogenicity of AML subclones. Conversely, a higher level of FC-MRD at all timepoints was strongly associated with lower survival at one year in AML but not B-ALL. Exploratory work on the longitudinal patterns of FC-MRD over a one-year period suggests that consecutive FC-MRD results may be more valuable than a singular reading and provide additional prognostic information that can be combined with ELN risk.

## Figures and Tables

**Table 1:** Descriptive characteristics of the 47 participants with B lymphoblastic leukaemia (B-ALL). Data displayed as *n* (%) unless otherwise indicated.

		*n* (%)
Age		
	Median (IQR)	51 (33.5–60.5)
Gender		
	Female	18 (35%)
	Male	29 (62%)
Treatment		
	Chemotherapy	32 (68%)
	AlloSCT	9 (19%)
	Surveillance	2 (4%)
Chemotherapy		
	Multimodal chemotherapy	19 (59%)
	Blinatumomab	3 (9%)
	Maintenance	10 (32%)
Conditioning regimen		
	Reduced-intensity conditioning	4 (44%)
	Myeloablative conditioning	5 (56%)
Donor type		
	Matched unrelated donor	3 (33%)
	Haploidentical donor	2 (22%)
	Sibling donor	4 (45%)
Genetics		
	t(9;22) (*BCR-ABL1*)	19 (53%)
	*IGH* gene rearrangement	17 (47%)
Flow MRD		
	Positive	12 (26%)
	Negative	33 (70%)
	Indeterminate	2 (4%)

AlloSCT—allogeneic stem cell transplant; MRD—measurable residual disease. Note: There are missing data for treatment (*n* = 4).

**Table 2:** Descriptive characteristics of the 87 participants with acute myeloid leukaemia (AML). Data displayed as *n* (%) unless otherwise indicated.

		*n* (%)
Age		
	Median (IQR)	60 (47.5–69.0)
Gender		
	Female	42 (48%)
	Male	45 (52%)
Treatment		
	Chemotherapy	49 (56%)
	AlloSCT	29 (33%)
	Surveillance	7 (8%)
Chemotherapy regimens		
	Intensive chemotherapy	32 (37%)
	Hypomethylating agent-based	5 (6%)
	Low-dose cytarabine-based regimen	12 (14%)
Conditioning regimens		
	Reduced-intensity conditioning	19 (22%)
	Myeloablative conditioning	10 (11%)
Donor type		
	Matched unrelated donor	14 (16%)
	Haploidentical donor	11 (13%)
	Sibling donor	4 (5%)
Genetics		
	t(8;21) *(RUNX1-RUNX1T1)*	8 (9%)
	inv(16) *(CBFB-MYH11)*	2 (2%)
	*NPM1* mutations	18 (21%)
	t(9;22) *(BCR-ABL1)*	1 (1%)
ELN risk		
	Favourable	25 (29%)
	Intermediate	23 (26%)
	Adverse	29 (33%)
	Other	6 (7%)
Flow MRD		
	Positive	44 (51%)
	Negative	23 (26%)
	Indeterminate	20 (23%)

AlloSCT—allogeneic stem cell transplant; MRD—measurable residual disease. Note: There are missing data for treatment (*n* = 2) and ELN risk (*n* = 4).

**Table 3:** Comparison of B-lymphoblastic leukaemia (B-ALL) measurable residual disease by flow cytometry (FC-MRD) and molecular (Mol-MRD) methodologies using a 0.01% cutoff. Percentages are calculated within each row.

Mol-MRD	FC-MRD				Total
	Positive > 5% (Relapse)	Positive	Indeterminate	Negative	
**Positive**	3 (12%)	10 (38%)	1 (4%)	12 (46%)	26 (100%)
**Negative**	0 (0%)	6 (8%)	8 (11%)	61 (81%)	75 (100%)
**Total**	3 (3%)	16 (16%)	9 (9%)	73 (73%)	101 (100%

**Table 4:** Comparison of acute myeloid leukaemia (AML) measurable residual disease (MRD) by flow cytometric (FC-MRD) and molecular (Mol-MRD) methods using a 0.01% cutoff. Percentages are calculated within each row.

Mol MRD	FC-MRD				Total
	Positive > 5% (Relapse)	Positive	Indeterminate	Negative	
**Positive**	1 (3%)	10 (30%)	8 (24%)	14 (43%)	33 (100%)
**Negative**	0 (0%)	16 (32%)	4 (8%)	30 (60%)	50 (100%)
**Total**	1 (1%)	26 (31%)	12 (15%)	44 (53%)	83 (100%)

**Table 5:** Association of B-ALL FC-MRD results with patient survival at 12 months at different timepoints.

Time of MRD Result	Survival	*n*	Median	*p*
Baseline	Alive	36	0	0.487
	Deceased	4	0.01	
3 months	Alive	25	0	0.014
	Deceased	2	0.17	
6 months	Alive	24	0	0.292
	Deceased	2	0.54	
9 months	Alive	20	0	0.18
	Deceased	3	8.4	

**Table 6:** Association of AML FC-MRD results with patient survival at 12 months at different timepoints.

Time of MRD Result	Survival	*n*	Median	*p*
Baseline	Alive	63	0.03	<0.001
	Deceased	13	1.39	
3 months	Alive	45	0.01	0.038
	Deceased	4	6.35	
6 months	Alive	45	0.01	0.017
	Deceased	5	10	
9 months	Alive	36	0	0.052
	Deceased	2	13.18	

## Data Availability

The data presented in this study are available on request from the corresponding author. The data are not publicly available due to privacy and ethical concerns.

## Supplementary Materials

The following supporting information can be downloaded at: https://www.mdpi.com/2072-6694/15/20/5064/s1, Figure S1: example of a case with positive measurable residual disease (MRD). 1A: Diagnostic sample showing the leukaemia-associated immunophenotype (LAIP). Blasts express CD34 (dim), CD117, CD13 (dim), HLA-DR, CD33 (dim/negative) and CD38. 1B: Subsequent sample showing MRD with a similar phenotype to the LAIP, except for brighter CD33 expression. The MRD accounted for 0.9% of CD45^+^ cells, Figure S2: example of a case with negative measurable residual disease (MRD). 1A: Diagnostic sample showing the leukaemia-associated immunophenotype (LAIP). Blasts express CD34 (dim), CD117, CD13 (bright), HLA-DR (dim), CD33 (bright) and CD38. 1B: The subsequent sample shows the absence of MRD based on the LAIP and no population with aberrant marker expression. The background shows normal maturation patterns for myeloid precursors, Table S1: monoclonal antibodies used for analysis of minimal residual disease in AML and BALL, Table S2: comparison of AML measurable residual disease by flow cytometric (FC-MRD) and molecular (Mol-MRD) methods grouped according to treatment received.
