# Supplementary material for: Measurable Residual Disease (MRD) by Flow Cytometry in Adult B-Acute Lymphoblastic Leukaemia (B-ALL) and Acute Myeloid Leukaemia (AML): Correlation with Molecular MRD Testing and Clinical Outcome at One Year
Source: Cancers (Basel). 2023 Oct 19;15(20):5064. doi: 10.3390/cancers15205064 (PMC10605425; doi:10.3390/cancers15205064)
Supplement: Supplementary file 1 [file cancers-15-05064-s001.zip › cancers-2639812-supplementary.pdf]

Supplementary information

Supplementary Table S1: Monoclonal antibodies used for analysis of minimal residual disease in AML and BALL.

Antibody	Clone	Antibody	Clone
**Myeloid tube**		**B lymphoblastic tube**	
CD10-FITC	W8E7	CD58-FIC	1C3 (AICD58.6)
CD13-PE	L138/Leu-M7)	CD10-PE	HI10a
CD19-PE-CF594	HIB19	CD22-ECD	SJ10
CD117-PC5.5	104D2D1	CD34-PE-Cy7	8G12
CD34-PC7	8G12	CD19-APC	HIB19
CD38-AF700	HIT2	CD38-AF700	HIT2
HLA-DR-APC-Vio770	AC122	CD20-APC-Vio770	L20
CD7-V450	M-T701	CD13-BV421	WM15
CD45-KO	J33	CD33-BV421	WM53
**Monocytic tube**		CD45-KO	J33
CD64-FITC	10.1		
CD14-PE	RMO52		
CD4-PE-CF594	RPA-T4		
CD117-PC5.5	104D2D1		
CD34-PC7	8G12		
CD38-AF700	HIT2		
HLA-DR-APC-Vio770	AC122		
CD15-V450	MMA		
CD45-KO	J33		

Supplementary Table S2: Comparison of AML measurable residual disease by flow cytometric (FC-MRD) and molecular (Mol-MRD) methods grouped according to treatment received.

		FC-MRD				
**Treatment**	**Mol-MRD**	**Positive**	**Indeterminate**	**Negative**	**Total**	**p**
**Current active treatment**	**Positive**	8	6	3	17	p=0.018
	**Negative**	5	1	10	16	
	**Total**	13	7	13	33	
**Post-transplant**	**Positive**	0	1	6	7	p=0.128
	**Negative**	9	3	11	23	
	**Total**	9	4	17	30	
**Surveillance**	**Positive**	2	1	5	8	p=0.528
	**Negative**	2	0	7	9	
	**Total**	4	1	12	17	

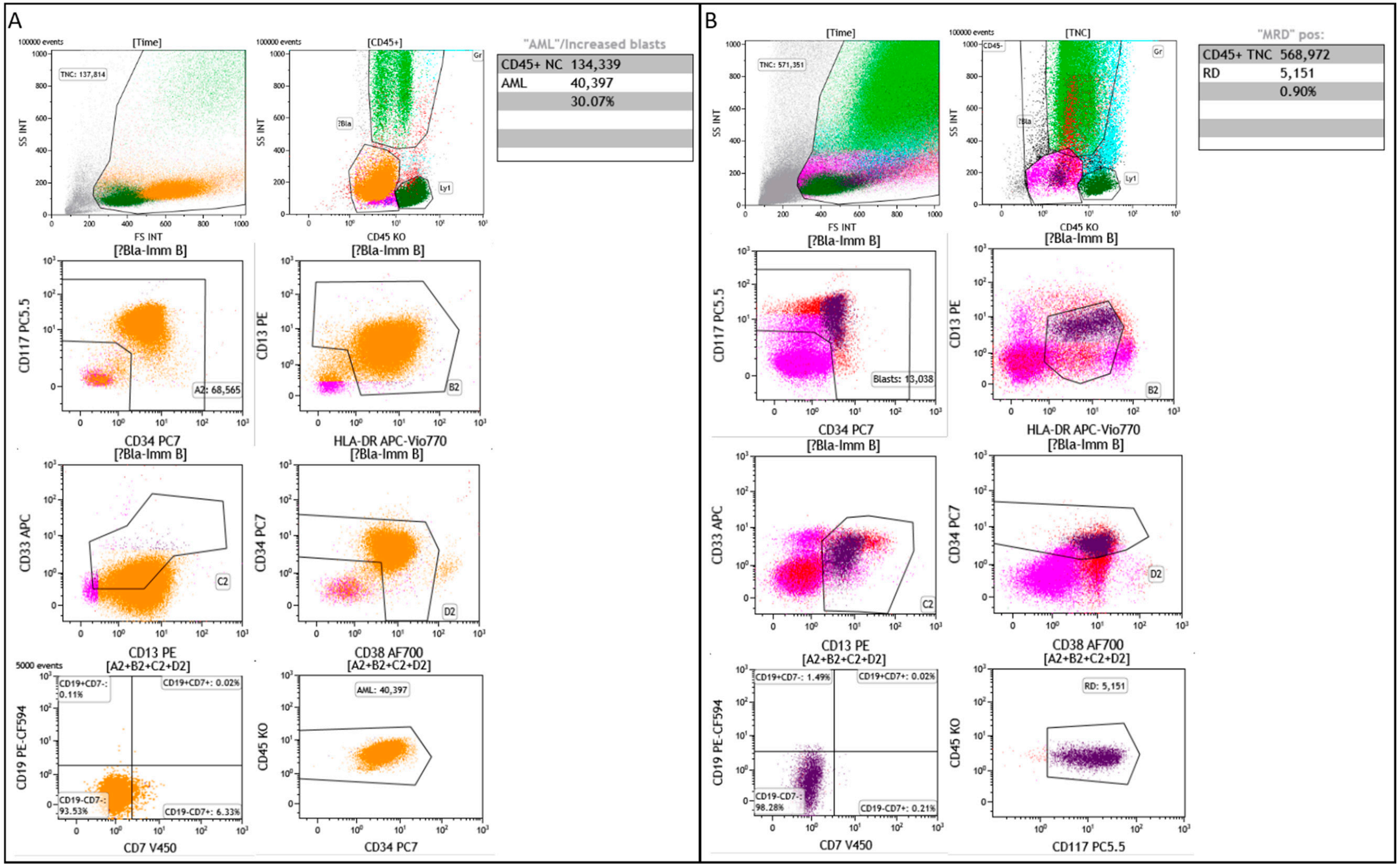

Supplementary Figure S1: Example of a case with positive measurable residual disease (MRD). 1A: Diagnostic sample showing the leukaemia associated immunophenotype (LAIP). Blasts express CD34 (dim), CD117, CD13(dim), HLA-DR, CD33(dim/negative) and CD38. 1B: Subsequent sample showing MRD with a similar phenotype to the LAIP, except for brighter CD33 expression. The MRD accounted for 0.9% of CD45^{+} cells.

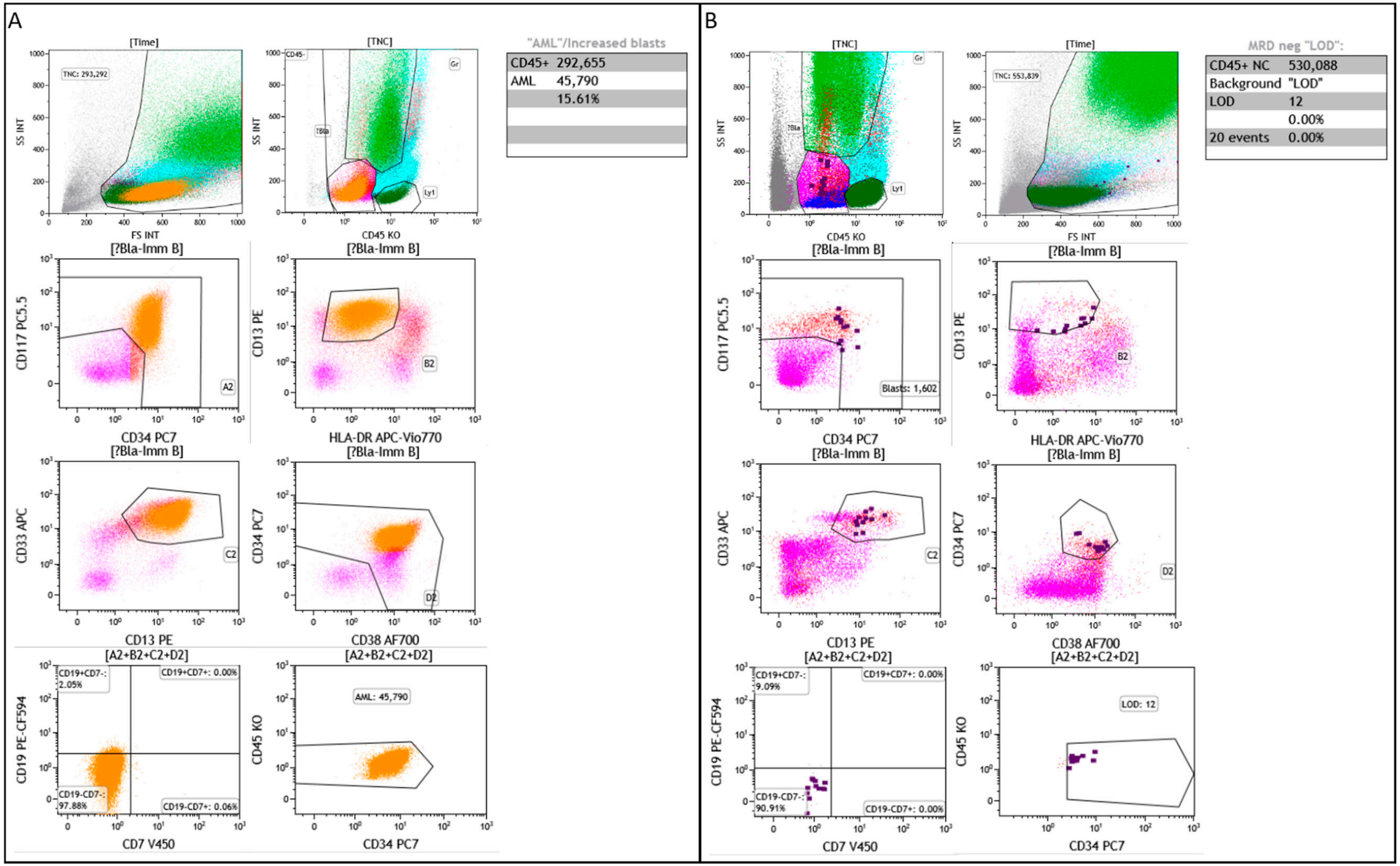

Supplementary Figure S2: Example of a case with negative measurable residual disease (MRD). 1A: Diagnostic sample showing the leukaemia associated immunophenotype (LAIP). Blasts express CD34 (dim), CD117, CD13(bright), HLA-DR(dim), CD33(bright) and CD38. 1B: Subsequent sample shows absence of MRD based on the LAIP and no population with aberrant marker expression. The background shows normal maturation patterns for myeloid precursors.
